# Anti-metastatic effect of GV1001 on prostate cancer cells; roles of GnRHR-mediated Gαs-cAMP pathway and AR-YAP1 axis

**DOI:** 10.1186/s13578-021-00704-3

**Published:** 2021-11-07

**Authors:** Ji Won Kim, Miso Park, Suntae Kim, Sung Chul Lim, Hyung Shik Kim, Keon Wook Kang

**Affiliations:** 1 Division of Hematology and Medical Oncology, University of California, San Francisco, CA 94143 USA; 2 College of Pharmacy and Research Institute of Pharmaceutical Sciences, Seoul National University, Seoul, 08826 Republic of Korea; 3 Department of Pathology, College of Medicine, Chosun University, Gwangju, 61452 Republic of Korea; 4 College of Pharmacy, Sungkyunkwan University, Suwon, 16419 South Korea

**Keywords:** GV1001, Prostate cancer, GnRHR, Gαs-cAMP, AR, YAP1, Migration

## Abstract

**Background:**

Gonadotropin-releasing hormone receptor (GnRHR) transmits its signal via two major Gα-proteins, primarily Gαq and Gαi. However, the precise mechanism underlying the functions of Gαs signal in prostate cancer cells is still unclear. We have previously identified that GV1001, a fragment of the human telomerase reverse transcriptase, functions as a biased GnRHR ligand to selectively stimulate the Gαs/cAMP pathway. Here, we tried to reveal the potential mechanisms of which GV1001-stimulated Gαs-cAMP signaling pathway reduces the migration and metastasis of prostate cancer (PCa) cells.

**Methods:**

The expression of epithelial-mesenchymal transition (EMT)-related genes was measured by western-blotting and spheroid formation on ultra-low attachment plate was detected after GV1001 treatment. In vivo Spleen-liver metastasis mouse model was used to explore the inhibitory effect of GV1001 on metastatic ability of PCa and the transwell migration assay was performed to identify whether GV1001 had a suppressive effect on cell migration in vitro. In order to demonstrate the interaction between androgen receptor (AR) and YAP1, co-immunoprecipitation (co-IP), immunofluorescence (IF) staining, chromatin immunoprecipitation (ChIP) were performed in LNCaP cells with and without GV1001 treatment.

**Results:**

GV1001 inhibited expression of EMT-related genes and spheroid formation. GV1001 also suppressed in vivo spleen-liver metastasis of LNCaP cells as well as cell migration in vitro. GV1001 enhanced the phosphorylation of AR and transcription activity of androgen response element reporter gene through cAMP/protein kinase A pathway. Moreover, GV1001 increased Ser-127 phosphorylation of YAP1 and its ubiquitination, and subsequently decreased the levels of AR-YAP1 binding in the promoter region of the CTGF gene. In contrast, both protein and mRNA levels of NKX3.1 known for tumor suppressor gene and AR-coregulator were upregulated by GV1001 in LNCaP cells. YAP1 knockout using CRISPR/Cas9 significantly suppressed the migration ability of LNCaP cells, and GV1001 did not affect the cell migration of YAP1-deficient LNCaP cells. On the contrary, cell migration was more potentiated in LNCaP cells overexpressing YAP5SA, a constitutively active form of YAP1, which was not changed by GV1001 treatment.

**Conclusions:**

Overall, this study reveals an essential role of AR-YAP1 in the regulation of PCa cell migration, and provides evidence that GV1001 could be a novel GnRHR ligand to inhibit metastasis of PCa via the Gαs/cAMP pathway.

**Supplementary Information:**

The online version contains supplementary material available at 10.1186/s13578-021-00704-3.

## Background

Gonadotropin-releasing hormone (GnRH), a decapeptide hormone released from the hypothalamus, is the central activator of the reproductive hormonal network [1, 2]. GnRH receptor (GnRHR) is a G-protein-coupled cell surface receptor (GPCR) with seven-transmembrane-spanning domains connected by extracellular and intracellular loops [3, 4]. As one of the GPCR families, GnRHR transduces the intracellular GPCR signals via multiple heterotrimeric G proteins [5, 6]. In pituitary gonadotropes, GnRHR primarily interacts with Gαq and exerts reproductive effects such as synthesis and secretion of pituitary gonadotropin hormones, luteinizing hormone (LH), and follicle-stimulating hormone (FSH) [7–9]. Conversely, it has been demonstrated that coupling of the GnRHR to Gαi abolishes neuronal function and hormonal secretion of GnRH [10]. In many types of human tissues and immortalized cell lines, it has been proposed that GnRHR interacts with different G-proteins to mediate the diverse physiological effects. Protein or mRNA expression of GnRH and GnRHR was detected not only in pituitary cells but also in extra-pituitary cells; ovarian, breast, prostate tissues, and various cancer cell lines including breast (MCF-7 and MDA-MB-468) and prostate cancer (PCa) cells (PC-3 and LNCaP) [11–14]. Several reports suggest that GnRHR expressed in cancer cells directly contributes to cancer progression, and GnRH analogs exert direct anti-cancer actions by modulating Gα subunits. GnRH analogs induced apoptosis of several prostate cancer cells presumably via Gαi coupling [15–19]. Also, GnRH analogs counteracted the tumor growth of androgen receptor (AR)-negative DU145 xenografts, further supporting their direct and reproductive system-independent anti-cancer effects [20]. Based on the evidence, cancer cell-expressing GnRHR is considered as a novel receptor-targeting anti-cancer strategy.

GV1001, a 16 amino acid peptide derived from the human telomerase reverse transcriptase (hTERT), has been proposed as a cancer vaccine to boost immune responses of CD8 and CD4 T cells [21]. We have recently demonstrated that GV1001 functions as a GnRHR ligand and selectively stimulates the Gαs-cAMP pathway [22]. Although it has been proposed that GnRH analogs regulate not only well-established Gαq but also Gαs pathway in several extra-pituitary cells [23–25], pharmacological effects of Gαs-coupled GnRHR activation has not been studied in cancer cells. In an attempt to elucidate the underlying molecular mechanisms of anti-migration and metastatic effects of GV1001 in PCa, we investigated the functions of Gαs-cAMP signaling under GV1001-stimulated GnRHR activation, focusing on AR and its coregulator, yes-associated protein1 (YAP1).

## Materials and methods

### Materials

Antibodies recognizing YAP1, phospho-YAP1 (Ser-127 p-YAP1)(Cell Signaling Technology, Beverly, MA, USA), matrix metalloproteinase2 (MMP2), slug, twist, vimentin, AR, NK3 Homeobox 1 (NKX3.1), Myc, connective tissue growth factor (CTGF, Santa Cruz Biotechnology, Santa Cruz, CA, USA), E-cadherin, N-cadherin (BD Biosciences, San Jose, CA, USA), phospho-AR (ser-81 p-AR)(Milipore, Billerica, MA, USA), and ß-actin, GAPDH (Sigma-Aldrich, St. Louis, MO, USA) were used for immunoblottings. Horseradish peroxidase (HRP)-conjugated donkey anti-rabbit and anti-mouse IgGs were obtained from Cell Signaling Technology. GV1001 peptide was supplied by GemVax & KAEL (Seongnam, Kyeonggi-do, South Korea). Leuprolide acetate (LA) and cetrorelix acetate (CA) were synthesized by AnyGen (Gwangju, South Korea). KT5720 (Cat# K3761), forskolin (Cat# F6886), dihydrotestosterone (Cat# A8380), flutamide (Cat# F9397), pertussis toxin (Cat# P2980) and SQ22536 (Cat# S153) were purchased from Sigma-Aldrich (St. Louis, MO, USA). KH7 (Cat# 13243, Cayman Chemical, Ann Arbor, MI, USA) and 1,2-Bis(2-aminophenoxy) ethane-N,N,N’,N’-tetraacetic acid tetrakis(acetoxymethyl ester, BAPTA/AM)(Biovision, Mountain View, CA, USA) were used as signaling inhibitors for Gα-proteins.

### Cell culture and establishment of stably GnRHR-overexpressing HEK293 cells

LNCaP (androgen receptor positive human PCa cell line) cells were cultured in RPMI-1640 medium with 10% fetal bovine serum (Hyclone, Logan, UT, USA) and 1% penicillin/streptomycin (100 U/ml, Hyclone). YAP1 knockout LNCaP cells were established by CRISPR/Cas9 gene editing system. U6-YAP1/Cas9-2A-RFP plasmid or CRISPR Universal Negative Control plasmid were transfected with LNCaP cells and the red fluorescence protein (RFP)-positive cells were further sorted by FACS Aria II flow cytometer (BD Biosciences, San Jose, CA, USA). HEK293 cells were cultured in Dulbecco's modified Eagle's medium (DMEM) with 10% fetal bovine serum and 1% penicillin/streptomycin. All cultures were maintained in a humidified 5% CO_2_ environment at 37 °C. To obtain GnRHR-overexpressing HEK293 (HEK293-GnRHR) cells, transfection of pcDNA3.1(+)-GnRHR vector was performed by using Lipofectamine 2000 as specified by the manufacturer's instruction (Invitrogen, Carlsbad, CA, USA). Geneticin-resistant colonies were selected by G418 (800 μg/ml, Thermo Fisher Scientific, Waltham, MA, USA) treatment. HEK293-pcDNA3.1(+) vector was also used as mock-transfection group.

### Establishment of AR and YAP1 knockout cells

AR- or YAP1-knockout LNCaP cells were established by using U6-AR/Cas9-2A-RFP plasmid (HS0000000952), U6-YAP1/Cas9-2A-RFP plasmid (HS0000121498) or CRISPR Universal Negative Control plasmid (Sigma-Aldrich). Lipofectamine 2000 (Thermo Fisher Scientific) was used as the manufacturer’s protocol. 48 h after transfection, AR or YAP1 knockout cells were selected. For the selection of RFP-positive-knockout cells, the transfected LNCaP cells were sorted using BD FACS Aria II flow cytometer (BD Biosciences, San Jose, CA, USA).

### Western blot analysis

After washing with PBS, cells were lysed with lysis buffer containing 20 mM Tris–Cl (pH 7.5), 1% Triton X-100, 137 mM sodium chloride, 10% glycerol, 2 mM EDTA, 1 mM sodium orthovanadate, 25 mM ß-glycerophosphate, 2 mM sodium pyrophosphate, 1 mM phenylmethylsulfonylfluoride and 1 μg/ml leupeptin. The cell lysates were centrifuged at 13,000×*g* for 15 min to remove insoluble material, the supernatants were fractionated using 10, 12 or 15% separating polyacrylamide gel, and electrophoretically transferred to nitrocellulose membranes, which were incubated overnight at 4 °C with one of the primary antibodies. HRP-conjugated anti-IgG antibodies were used as the secondary antibodies. The nitrocellulose papers were developed using an ECL chemiluminescence system (Milipore, Billerica, MA, USA). For ECL chemiluminescence detection, LAS-3000 mini system (Fujifilm, Tokyo, Japan) was used.

### Reporter gene assay

LNCaP, HEK293-pcDNA3.1( +) and HEK293-GnRHR cells (1 × 10^5^ cells/well) were cultured in 48-well plates, then transfected with luciferase reporter plasmid containing cAMP response element (CRE) or androgen response element (ARE). In order to evaluate the transcriptional activity of YAP1, YAP1 knockout- and wildtype-LNCaP cells were cultured in 48-well plates and transfected with YAP/TAZ-responsive luciferase reporter plasmid (TEAD reporter). CRE-luciferase plasmid was purchased from Stratagene (La Jolla, CA, USA) and ARE-luciferase plasmid) [61] was kindly donated from Dr. Young Joo Lee (Department of Bioscience and Biotechnology, Sejong University, Republic of Korea. Lipofectamine 2000 (Thermo Fisher Scientific) was used per the manufacturer’s protocol. The transfected cells were exposed to compounds for 24 h, and the promoter activity was measured using a dual-luciferase reporter assay system (Promega, Madison, WI, USA). The firefly and *hRenilla* luciferase activities in the cell lysates were measured using a luminometer (LB960, Berthold Technologies, Bad Wildbad, Germany). The relative luciferase activities were calculated by normalizing the promoter-driven firefly luciferase activity to phRL-SV (*hRenilla*) luciferase (Promega, Madison, WI, USA).

### Spheroid formation assay

Cells were detached into a single-cell suspension in RPMI medium containing 10% FBS at a density of 5 × 10^3^ cells/mL. The suspended cells (1 × 10^3^ cells/well) were seeded and allowed to form spheroids for 4 days onto ultra-low-attachment (ULA) plates (Costar, Corning Inc., Corning, NY, USA). The number of spheroids (average diameter > 100 μm, Scale bar = 300 μm) was determined.

### Transwell migration assay

LNCaP cells were seeded in the upper chamber of the transwell plate (Essen BioScience, Ann Arbor, MI, USA) and the lower chamber was filled with 200 μl serum-containing media. The cells were incubated at 37ºC for 24 h and the migrated cell numbers were counted using IncuCyte ZOOM live-cell analysis system (Essen BioScience, Ann Arbor, MI, USA).

### Immunocytochemistry

LNCaP cells were cultured overnight on coverslips. After treatment with 10 μM GV1001 or vehicle, the cells were fixed with 4% paraformaldehyde and blocked with 1% bovine serum albumin at room temperature for 1 h. The cells were incubated with rabbit monoclonal YAP/TAZ or phospho-YAP antibody (1:200 dilution) in 0.1% Tween20-containing PBS at 4 °C overnight. After twice washing with PBS, the coverslips were incubated with goat anti-rabbit Alexa Fluor-488 or -568 IgG (1:2000 dilution, Thermo Fisher Scientific, Waltham, MA, USA) at room temperature with ProLong Gold Antifade reagent with 4’,6-diamidino-2-phenylindole (DAPI, Invitrogen, Carlsbad, CA, USA). Images were obtained using SP8-STED Confocal Microscope System (Leica Microsystems, Heidelberg, Germany).

### Quantitative reverse transcription-polymerase chain reaction (qRT-PCR)

Total RNA was isolated from LNCaP cells using Trizol® reagent (Invitrogen, Carlsbad, CA, USA), and cDNA was synthesized by reverse transcriptase using an oligo(dT) primer. PCR was performed using the selective primers for human MMP2 (sense: 5′-CTTCCAAGTCTGGAGCGATGTG-3′, antisense: 5′-ATGAGCCAGGAGTCCGTCCTTA-3′), human AR (sense: 5′-AGGATGCTCTACTTCGCCCC-3′, antisense: 5′-ACTGGCTGTACATCCGGGAC-3′), human prostate specific antigen (PSA, sense: 5′-ACCAGAGGAGTTCTTGACCCCAAA-3′, antisense: 5′-CCCCAGAATCACCCGAGCAG-3′), human CTGF (sense: 5′-CAAGGGCCTCTTCTGTGACT-3′, antisense: 5′-ACGTGCACTGGTACTTGCAG-3′), human NKX3.1 (sense: 5′-GCCGCACGAGCAGCCAGAGACA-3′, antisense: 5′-TTCAGGGCCGGCAAAGAGGAGTG-3′), human GAPDH (sense: 5′-AAGGCTGAGAACGGGAAG-3′, antisense: 5′-GCCCCACTTGATTTTGGA-3′). The SYBR Green real-time PCR amplifications were conducted with MiniOpticon real time PCR detection system (Bio-Rad laboratories Inc., Hercules, CA, USA).

### Chromatin immunoprecipitation (ChIP) assay

ChIP assays were performed according to the EZ-ChIP kit (Milipore, Bedford, MA, USA). An anti-AR antibody (Cat# sc-819, Santa Cruz Biotechnology, Santa Cruz, CA, USA) was used to precipitate DNA–protein complexes. ChIP-derived DNA was quantified using qRT-PCR with SYBR Green real-time PCR Mastermix (Bio-Rad laboratories Inc., Hercules, CA, USA). The primers specific for the promoters were as follows: human PSA (sense: 5′-GCTAGCACTTGCTGTTCTGC-3′, antisense: 5′-GGGATCAGGGAGTCTCACAA-3′), human CTGF (sense: 5′-TGTGCCAGCTTTTTCAGACG-3′, antisense: 5′-TGAGCTGAATGGAGTCCTACACA-3′) and human NKX3.1 (sense: 5′-CCGAGCCAGAAAGGCACTTG-3′, antisense: 5′-CTTAGGGGTTTGGGGAAGCC-3′).

### Spleen-liver metastasis

5-Week-old male BALB/c-nu mice (Jung Ang Lab Animal Inc., Seoul, South Korea) were anaesthetized and an abdominal incision was made in the left flank and the spleen was isolated. 1 × 10^5^ LNCaP cells in 100 μl PBS were injected into the spleen with a 26-gauge needle, and the incision was closed with stitches. After 3 days, mice were divided into three groups: vehicle, GV1001 (10 mg/kg, daily, sc) and LA (0.1 mg/kg, daily, sc) treatment groups. After 4 weeks, the mice were sacrificed and the liver and spleen samples were analyzed by hematoxylin & eosin (H&E) staining. For pathological assessment, specimens were cut into 2 mm thick sections after formalin fixation. All abnormal regions (nodules or suspicious regions) were observed under a microscope, and the absolute metastatic tumor area was analyzed. Basically all abnormal regions were analyzed (3–4 slides per liver tissue) when nodules are found.

### Protein–protein interaction (PPI) network

The STRING database (http://string-db.org) [26] was used to analyze the protein–protein interactions. The STRING database was applied to predict the PPIs by DEGs, and the parameter of combined score > 0.4 was set as the threshold value for choosing significant interactions. The nodes in the PPI network were ranked by their connectivity degrees, which correspond to the number of interactions by other proteins.

### Statistics

Statistical analysis was performed using one-way ANOVA and Tukey’s post hoc multiple comparisons to determine the differences. The statistical significance was accepted at *P < 0.05 and **P < 0.01.

## Results

### Anti-migratory and anti-metastatic effects of GV1001 on human malignant PCa

**Fig. 1:**
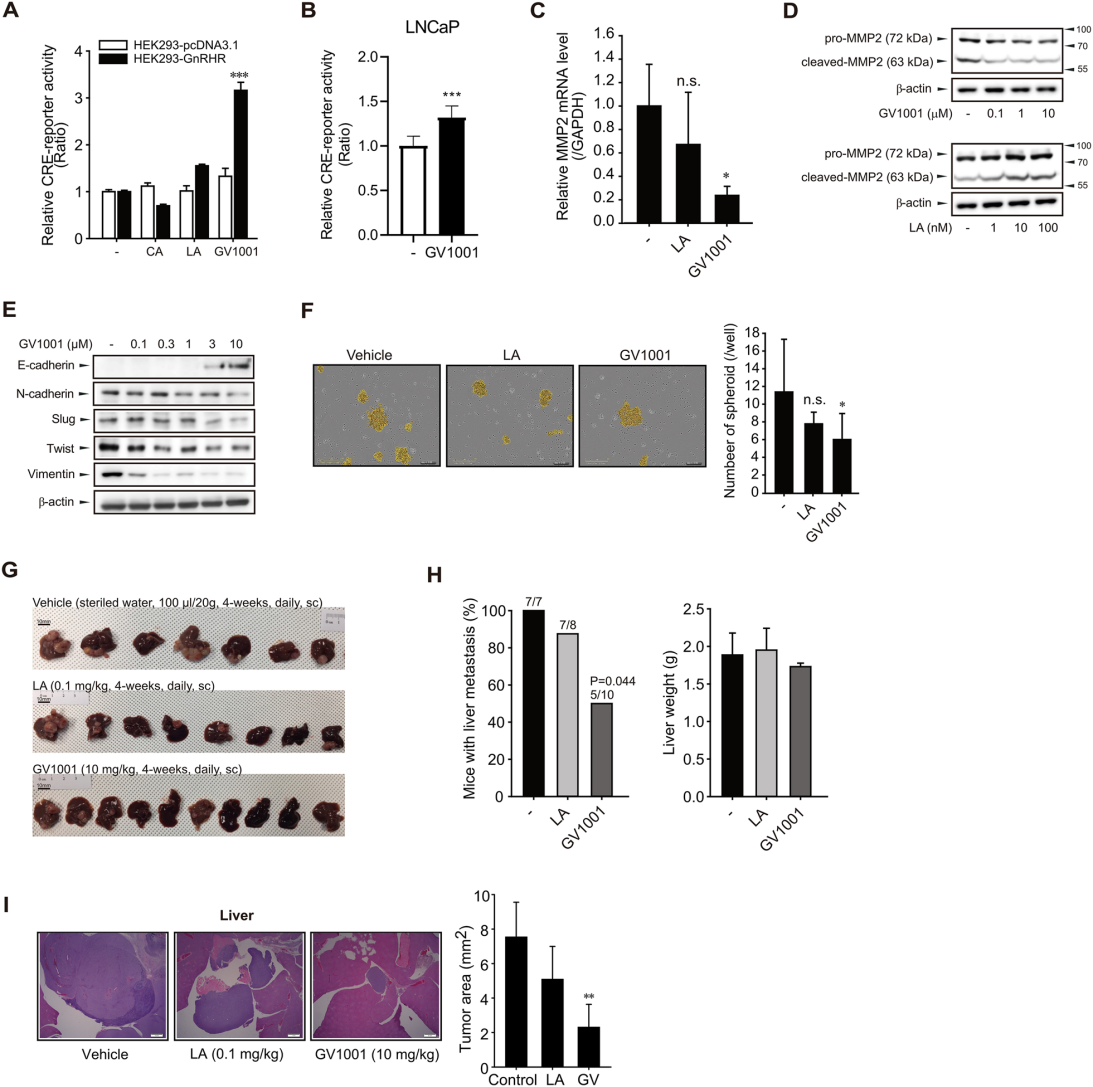
Effects of GV1001 on LNCaP-cell migration and tumor metastasis. **A** Effects of CA, LA, and GV1001 on CRE-reporter activity in HEK293-pcDNA3.1(+) and HEK293-GnRHR cells. The cells were exposed to CA (100 nM), LA (100 nM), or GV1001 (10 μM) for 24 h. Data represent means ± SD (n = 6, ***P < 0.005 vs vehicle-treated control). **B** Effect of 10 μM GV1001 on CRE-reporter activity in LNCaP cells. Data represent means ± SD (n = 6, ***P < 0.005 vs vehicle-treated control). **C** mRNA levels of MMP2. RT-qPCR was performed in LNCaP cells treated with LA (100 nM) or GV1001 (10 μM) for 24 h. **D** Determination of pro-MMP2 and cleaved-MMP2 proteins in LNCaP cells. LNCaP cells were incubated with or without GV1001 (0.1–10 μM) or LA (1–100 nM) for 24 h, and the cell lysates were subjected to immunoblotting using specific antibodies. **E** Protein expressions of EMT-related genes in LNCaP cells. LNCaP cells were incubated with GV1001 (0.1–10 μM) for 24 h. **F** Effects of GV1001 or LA on the spheroid formation of LNCaP cells. LNCaP cells were seeded in a 96-well ULA plate and treated with LA (100 nM) or GV1001 (10 μM) for 96 h. The number of spheroids (average diameter > 100 μm) was determined. Scale bar = 300 μm. Data represent means ± SD (n = 4, *P < 0.05 vs vehicle-treated control). **G**–**I** Spleen-liver metastasis in BALB/c-nude mice implanted with LNCaP cells into their spleens. After implantation, the mice were divided into 3 groups (n = 7–10) and then injected with vehicle (sterile water 100 μl, daily, sc), GV1001 (10 mg/kg, daily, sc) or LA (0.1 mg/kg, daily, sc) for 4 weeks. **G** Representative pictures of the metastatic liver tumors from each group are shown. Scale bar = 10 mm. **H** Statistical analysis of the incidence of liver metastatic colonization (the percentage of mice with metastatic tumor burden in the liver) and weights of tumor-bearing livers. Data represent means ± SD (n = 7–10, P = 0.044 vs vehicle-treated control, The P-value was calculated by two-sided Fisher exact test.). **I** Histological analysis of the metastasized-liver section. Data represent means ± SD (n = 7–10, **P < 0.01 vs vehicle-treated control)

We recently reported that Gαs-cAMP signaling is selectively activated by GV1001 binding to GnRHR, but not by LA, a representative GnRHR agonist [22]. We compared cAMP-dependent transcriptional activities of CA (a representative GnRHR antagonist), LA and GV1001 in GnRHR-overexpressing HEK293 cells. Among the three ligands, only GV1001 increased the reporter activity of the cAMP-responsive element, which confirms that GV1001 is a unique GnRHR ligand to selectively activate Gαs coupling (Fig. 1A). GV1001 also increased the CRE reporter activity in LNCaP cells (Fig. 1B) [22]. We then raised a question that which phenotype is under the control of cAMP increase by GnRHR/Gαs coupling. In our previous study, we revealed that the proliferation of PCa cells was slightly inhibited by both GV1001 and LA, but cell migration was only suppressed by GV1001 [22]. As we found the reduced mRNA levels of matrix metalloproteinase (MMP) 2 and MMP9 in tumor tissues from LNCaP xenografts injected with GV1001 [22], we further assessed both mRNA and protein expressions of MMP2 and MMP9 in LNCaP cells exposed to vehicle or GV1001. The MMP2 mRNA expression was significantly decreased by 10 μM GV1001, but MMP9 mRNA was not detected (data not shown) in LNCaP cells (Fig. 1C). Moreover, cleavage of MMP2 indicating the activation of MMP2 was also reduced by 10 μM GV1001 (Fig. 1D). From the above results and previous findings, we hypothesized that the anti-migratory effect of GV1001 is related to Gαs activation via GnRHR binding. To clarify whether the reduced cell motility by GV1001 is associated with the epithelial mesenchymal transition (EMT) process, we examined the levels of mesenchymal markers in LNCaP cells. We found that the protein levels of N-cadherin, vimentin, and the major transcription factors of EMT, slug and twist in LNCaP cells were significantly reduced by GV1001 (0.1–10 μM) (Fig. 1E). Since spheroid formation on a 3D-culture or ultra-low attachment (ULA) plate is a whole marker for EMT progress of cancer cells [27, 28], we then performed the spheroid formation assay. Spheroid formation on the ULA plate was significantly inhibited by 10 μM GV1001, but not by 100 nM LA (Fig. 1F). The data indicate that GV1001 inhibits migration of LNCaP cells presumably via targeting the EMT process. To determine the in vivo efficacy of GV1001, anti-metastasis activity was assessed in the spleen-liver metastasis model. GV1001 (10 mg/kg, daily sc)-injected group showed the lower incidence rate of liver metastasis (5/10, *P* value = 0.044) compared to vehicle (sterile water, daily sc) (7/7)- or LA (0.1 mg/kg, daily sc)-injected group, with no liver weight loss (Fig. 1G, H). Moreover, tumor area in liver tissues was also potently diminished in GV1001 injected mice compared to vehicle-injected mice (Fig. 1I).

### Inhibition of PCa cell migration by AR activation

**Fig. 2:**
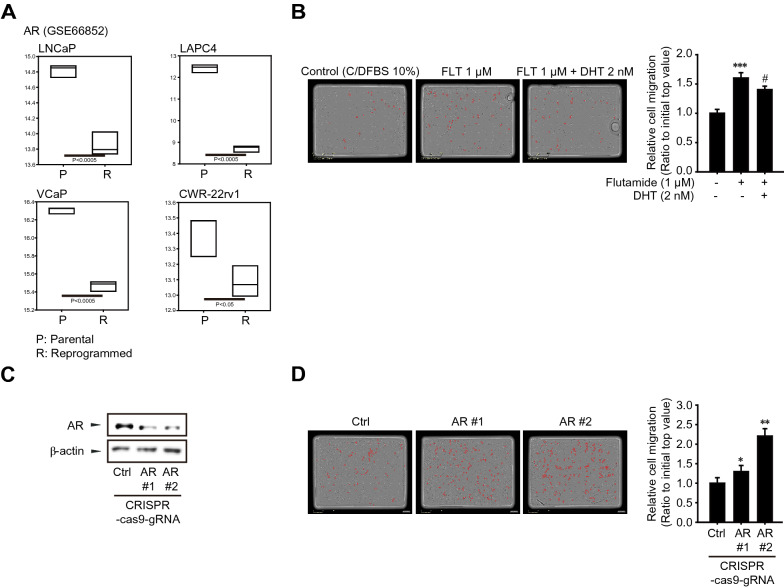
Role of AR activity in LNCaP cell migration. **A** Gene expression of AR in therapy-induced developmental reprogrammed-PCa cells (n = 3) as compared to parental PCa cell lines (GSE66852). Data were shown as box and whisker plots. Box, interquartile range (IQR); whiskers, 5–95 percentiles; and horizontal line within the box, median. **B** AR-mediated inhibition of LNCaP cell migration. LNCaP cells were treated with flutamide (AR antagonist, 1 μM) in the presence or absence of dihydrotestosterone (DHT, AR agonist, 2 nM) for 24 h. Migratory cell numbers were determined by the IncuCyte ZOOM live-cell analysis system. Data represent means ± SD (n = 4, ***P < 0.005 vs vehicle-treated control, ^#^P < 0.05 vs flutamide-treated group). **C** Development of AR knockout LNCaP cells by CRISPR/Cas9 system. Expression of AR was determined by immunoblot analysis. **D** Migration of AR knockout LNCaP cells. Migratory cell numbers were determined by IncuCyte ZOOM live-cell analysis system for 24 h. Data represent means ± SD (n = 4, *P < 0.05, **P < 0.01 vs vehicle-treated control)

EMT phenotype is closely correlated with AR signaling in castration-resistant PCa cells [29–31]. When we analyzed microarray data (GSE66852) of four different PCa cell lines, mRNA expressions of AR in all PCa cell lines were significantly decreased by developmental stem-transition reprogramming (Fig. 2A). We then examined the effects of AR activation on the migration of LNCaP cells. The number of migrated cells was significantly increased by 1 μM flutamide, AR antagonist compared to vehicle-treated cells, and the enhanced cell migration by flutamide was reversed by dihydrotestosterone, an active form of androgen (Fig. 2B). To confirm these results, we established AR knockout LNCaP cells using the CRISPR/Cas9 gene-editing system (Fig. 2C). The number of migrated AR knockout LNCaP cells was 2.2-fold higher than that of control cells (Fig. 2D). The data support the notion that AR activation negatively regulates the migratory capacity of AR-positive PCa cells.

### AR activation by GV1001 in PCa cells

**Fig. 3:**
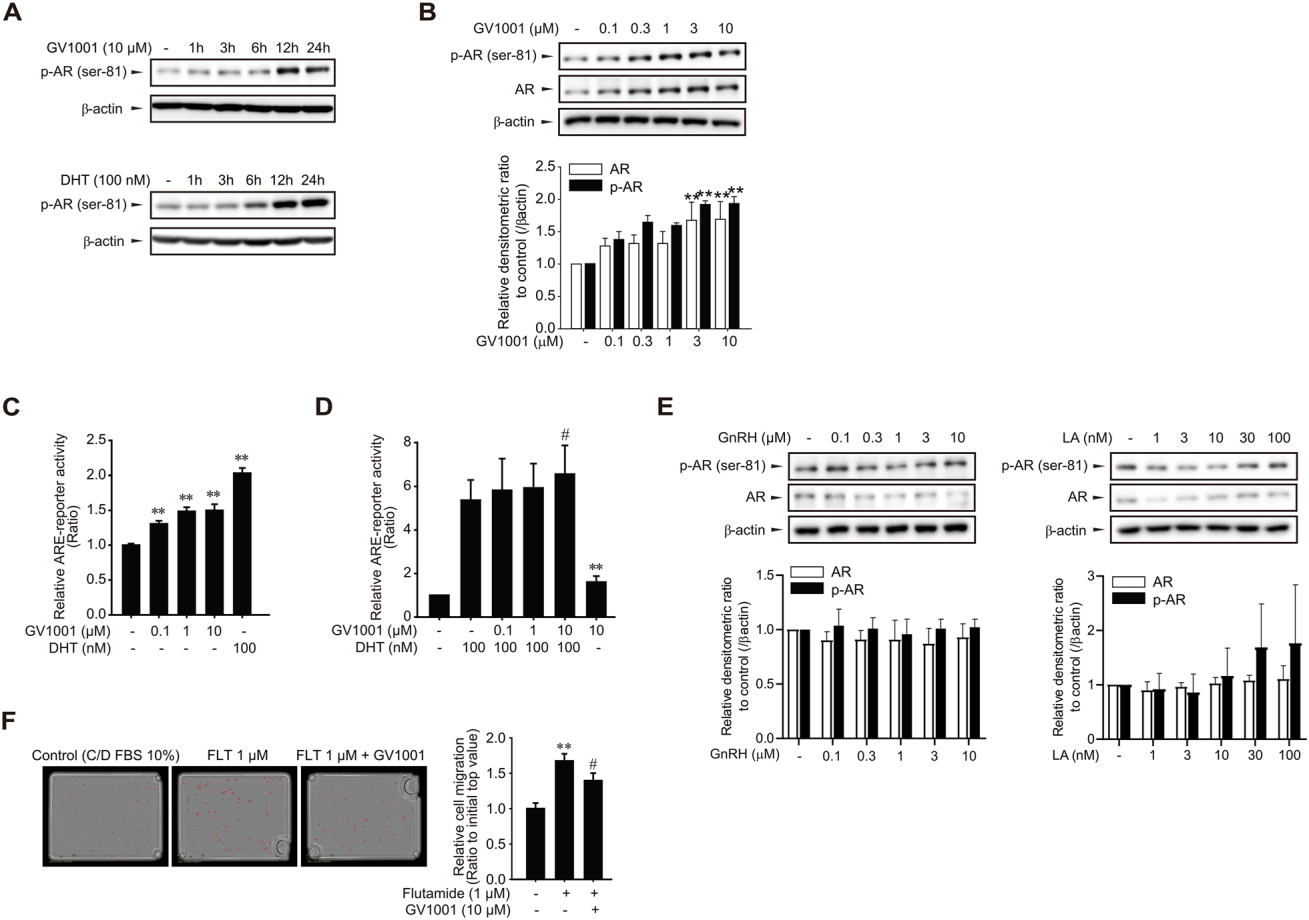
GV1001-induced AR activation in LNCaP cells. **A** Time-dependent AR phosphorylation (Ser-81) by dihydrotestosterone (DHT, 100 nM) or GV1001 (10 μM) in LNCaP cells. All of the results were confirmed by multiple experiments. **B** Total AR expression and AR phosphorylation in LNCaP cells treated with GV1001 (0.1–10 μM) for 24 h. **C** Effect of GV1001 on ARE-reporter activity. LNCaP cells were transiently transfected with ARE-luciferase plasmid (1 μg/ml) and the cells were exposed to DHT (100 nM) or GV1001 (0.1–10 μM) for 24 h. Luciferase activity was determined to assess ARE responsiveness. Data represent means ± SD (n = 6, **P < 0.01 vs vehicle-treated control). **D** An additive increase in ARE-reporter activity by GV1001 and DHT. GV1001 (0.1–10 μM) was simultaneously treated with dihydrotestosterone (100 nM) in LNCaP cells for 24 h. Data represent means ± SD (n = 8, **P < 0.01 vs vehicle-treated control, ^#^P < 0.05 vs DHT-treated group). **E** Expression and phosphorylation of AR by GnRH analogs. LNCaP cells were treated with GnRH (0.1–10 μM) or LA (1–100 nM) for 24 h. **F** Inhibitory effect of GV1001 on flutamide-stimulated LNCaP cell migration. LNCaP cells were treated with flutamide (1 μM) and GV1001 (10 μM) for 24 h. Migratory cell numbers were determined by the IncuCyte ZOOM live-cell analysis system. Data represent means ± SD (n = 4, **P < 0.01 vs vehicle-treated control, ^#^P < 0.05 vs flutamide-treated group)

We determined Ser-81 phosphorylation AR in LNCaP cells to examine the involvement of AR activation in GV1001-mediated inhibition of cell migration. Notably, we observed that 10 μM GV1001increased the phosphorylation of AR 12 h after exposure, and the pattern was similar to dihydrotestosterone treatment (Fig. 3A). We also found that AR protein expression, as well as AR phosphorylation, concentration-dependently increased by GV1001 (0.1–10 μM) (Fig. 3B). To assess the effect of GV1001 on AR-mediated transcriptional activity, androgen response element (ARE)-driven reporter activity was determined. Although the induction intensity was lower than dihydrotestosterone, GV1001 (0.1–10 μM) increased the ARE-reporter activity (Fig. 3C). Moreover, the additive increase in ARE-reporter activity was seen in LNCaP cells cotreated with GV1001 (10 μM) and 100 nM dihydrotestosterone (Fig. 3D), which imply that GV1001 acts as a ligand-independent AR activator. Because we previously revealed that GV1001 is a novel ligand of GnRHR, AR phosphorylation effects were assessed by using classical GnRHR ligands. Both GnRH and LA, representative endogenous and synthetic GnRHR ligands mainly activating the Gαq pathway, did not cause any significant phosphorylation of AR (Fig. 3E). We next assessed the effect of GV1001 on flutamide-induced cell migration in LNCaP cells. The enhanced migration was significantly attenuated by 10 μM GV1001 (Fig. 3F). These results indicate that AR activation may contribute to the inhibitory effects of GV1001 on cell migration.

### Gαs/cAMP-dependent AR phosphorylation by GV1001

**Fig. 4:**
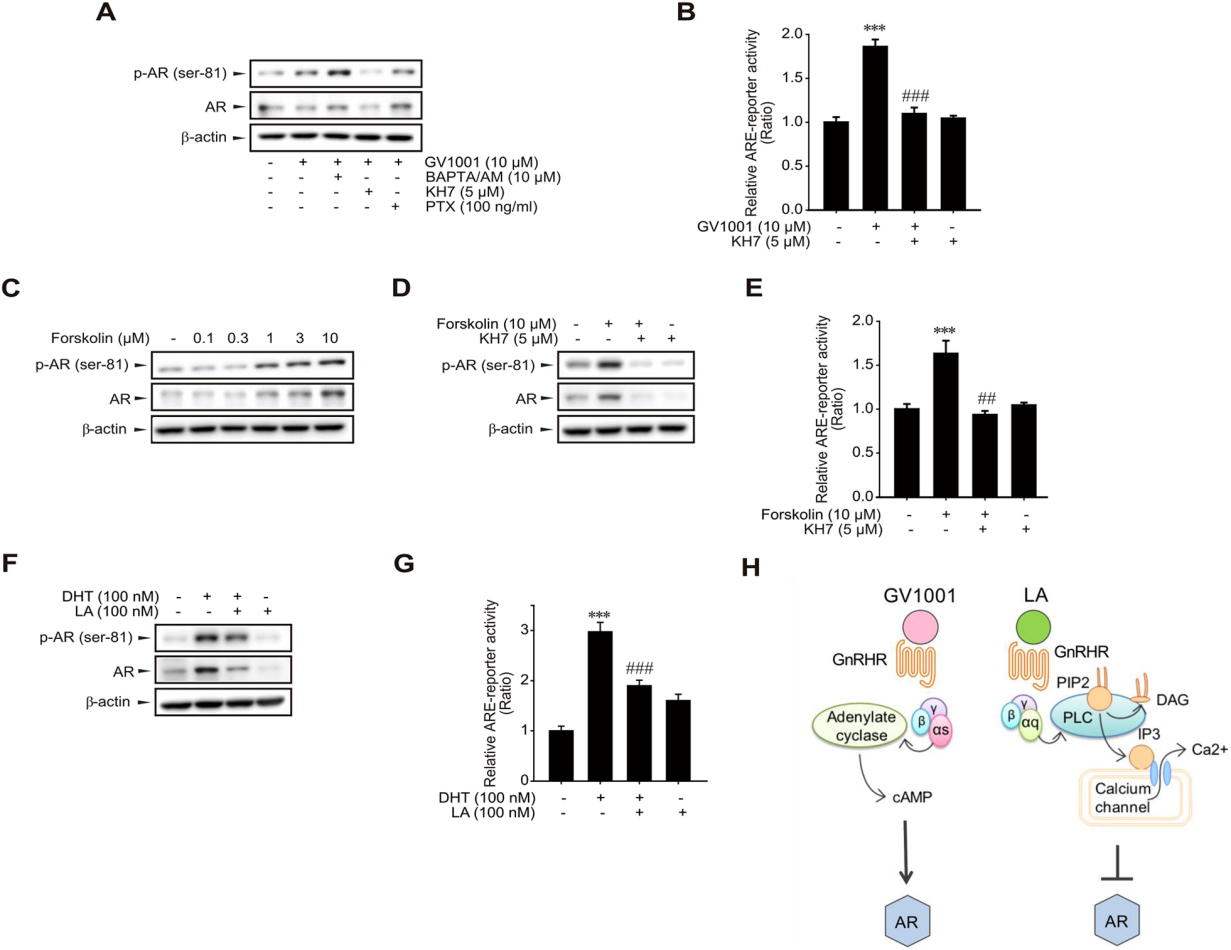
Involvement of Gαs-cAMP signaling pathway in GV1001-stimulated AR activation. **A** Effects of Gα signaling inhibitors on GV1001-stimulated AR phosphorylation in LNCaP cells. LNCaP cells were exposed to various Gαs inhibitors, a cell-permeable cytosolic calcium chelator BAPTA/AM (10 μM), a specific adenylyl cyclase inhibitor KH7 (5 μM) and an ADP-ribosyltransferase of Gαi, pertussis toxin (100 ng/ml) with 10 μM GV1001. All of the results were confirmed by multiple experiments. **B** Inhibitory effect of KH7 on GV1001-induced ARE-reporter activity. LNCaP cells were transiently transfected with ARE-luciferase plasmid (1 μg/ml) and the cells were exposed to KH7 (5 μM) and GV1001 (10 μM) for 24 h. Luciferase activity was determined to assess ARE responsiveness. Data represent means ± SD (n = 5, **P < 0.01 vs vehicle-treated control, ^###^P < 0.005 vs GV1001-treated group). **C** AR activation by forskolin. LNCaP cells were treated with forskolin, a cAMP activator (0.1–10 μM) for 24 h, and protein expression of AR and phosphorylated AR was assessed by immunoblotting. **D** Inhibitory effect of KH7 on forskolin-stimulated AR expression and phosphorylation. LNCaP cells were exposed to 5 μM KH7 and 10 μM forskolin for 24 h. **E** Inhibitory effect of KH7 on forskolin-induced ARE-reporter activity in LNCaP cells. LNCaP cells were transiently transfected with ARE-luciferase plasmid (1 μg/ml) and the cells were exposed to KH7 (5 μM) and forskolin (10 μM) for 24 h. Luciferase activity was determined to assess ARE responsiveness. Data represent means ± SD (n = 6, **P < 0.01 vs vehicle-treated control, ^##^P < 0.01 vs forskolin-treated group). **F**, **G** Inhibitory effect of LA on DHT-induced AR activation. **F** Inhibitory effect of LA on DHT-induced AR phosphorylation. LNCaP cells were pretreated with LA (100 nM) for 1 h and treated with DHT (100 nM) for 24 h. **G** Inhibitory effect of LA on DHT-induced ARE-reporter activity. LNCaP cells were transiently transfected with ARE-luciferase plasmid (1 μg/ml) and the cells were exposed to DHT (100 nM) and LA (100 nM) for 24 h. Luciferase activity was determined to assess ARE responsiveness. Data represent means ± SD (n = 6, **P < 0.01 vs vehicle-treated control, ^###^P < 0.005 vs DHT-treated group). **H** Hypothetical diagram for the differential effects of GV1001 and LA on AR

Although most GnRHR ligands preferentially activate Gαq-calcium signaling, our previous study demonstrated that GV1001 binding to GnRHR selectively activates Gαs-cAMP signaling [22]. To identify the upstream signaling for Ser-81 phosphorylation of AR, we used diverse inhibitors targeting G protein signaling. LNCaP cells were treated with 10 μM BAPTA/AM (a cell-permeable calcium chelator, Gαq inhibitor), 5 μM KH7 (a specific soluble adenylate cyclase inhibitor, Gαs inhibitor), 100 ng/ml pertussis toxin (PTX, an ADP-ribosylating toxin, an inhibitor of Gαi) in the presence or absence of 10 μM GV1001. KH7 blocked GV1001-stimulated AR phosphorylation (Fig. 4A). Moreover, KH7 almost completely suppressed ARE-driven reporter activity induced by 10 μM GV1001 (Fig. 4B). Vice versa, forskolin (0.1–10 μM), an adenylate cyclase activator, increased AR phosphorylation in LNCaP cells (Fig. 4C), and the enhanced AR phosphorylation and ARE reporter activity by 10 μM forskolin was inhibited by KH7 (Fig. 4D, E). As shown in Fig. 4A, cotreatment with BAPTA/AM and GV1001 potently increased AR phosphorylation. Thus, in order to determine whether Gαq-calcium signaling is linked to AR activity, we further tested the effect of LA, a representative Gαq-stimulating ligand of GnRHR on AR phosphorylation. Interestingly, 100 nM LA abrogated dihydrotestosterone-stimulated AR phosphorylation (Fig. 4F) and ARE-reporter activity (Fig. 4G). The data suggest that AR phosphorylation and its transcriptional activity are reciprocally controlled by GnRHR-coupled Gαs and Gαq (Fig. 4H).

### AR coregulator switching by GV1001

**Fig. 5:**
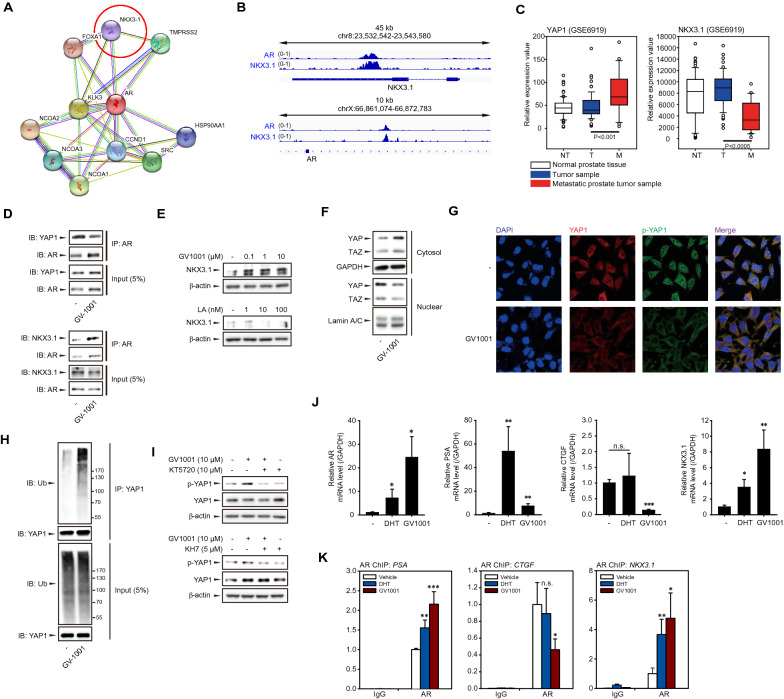
AR coregulator switching by GV1001. **A** Protein–protein interaction network analysis of predicted and reported functional partners of AR. Gene interactions were constructed using the STRING online database (http://string-db.org/). Network nodes represent proteins and the edges represent protein–protein associations. **B** Binding probability of AR and NKX3.1 at the transcription initiation site of the human NKX3.1 and AR genes. Experimental data from the chromatin immunoprecipitation (ChIP) atlas (http://chip-atlas.org/) are shown using Integrative Genomics Viewer (IGV) software. **C** Expressions in normal prostate tissues, primary prostate tumor tissues, and metastatic prostate tumor tissues from prostate cancer patients (n = 164) (GSE6919). DATA were shown as box and whisker plots. Box, interquartile range (IQR); whiskers, 5–95 percentiles; and horizontal line within the box, median. **D** Protein interaction of YAP1 or NKX3.1 with AR. LNCaP cells were treated with GV1001 (10 μM) for 24 h, and reciprocal immunoprecipitation and immunoblotting were performed. All of the results were confirmed by multiple experiments. **E** GV1001 or LA-induced NKX3.1 expression. LNCaP cells were treated with GV1001 (0.1–10 μM) or LA (1–100 nM) for 24 h. **F** Cytoplasmic retention of YAP1 by GV1001. LNCaP cells were treated with GV1001 (10 μM) for 24 h, and subcellular fractionation and subsequent immunoblot analyses were performed. **G** Immunocytochemistry of YAP1 and phospho-YAP1 proteins in LNCaP cells. LNCaP cells were treated with 10 μM GV1001 for 4 h. Alexa Fluor 568 stained YAP1 (red), Alexa Fluor 488 stained phospho-YAP1 (green) and DAPI stained nuclei (blue). Magnification: 60X. **H** Ubiquitination induction by GV1001. LNCaP cell lysates were immunoprecipitated with YAP1 antibody and analyzed by immunoblotting with anti-ubiquitin antibody to detect polyubiquitinated YAP1. **I** Inhibitory effects of KT5720 (upper) and KH7 (lower) on GV1001-induced YAP1 phosphorylation. KT5720 (10 μM) or KH7 (5 μM) was added 1 h before 10 μM GV1001 treatment in LNCaP cells, and 24 h after total cell lysates were subjected to immunoblotting for phospho-YAP1 and YAP1. **J** mRNA levels of target genes for AR/coregulator complex. RT-qPCR was performed in LNCaP cells treated with DHT (100 nM) or GV1001 (10 μM) for 24 h. **K** ChIP-qPCR analysis of AR recruitment to the promoter region of PSA, CTGF or NKX3.1 gene. LNCaP cells were treated with DHT (100 nM), GV1001 (10 μM) for 24 h. Input and immunoprecipitated samples were analyzed by qPCR for PSA, CTGF, or NKX3.1 promoter region. ChIP-qPCR data are presented as a percentage of input. Data represent means ± SD (n = 6, **P < 0.01 vs vehicle-treated control)

The transcriptional activity of AR is modulated by numerous AR coregulators [32]. To explore the molecular mechanism by which AR activation suppresses PCa cell migration, we performed a network analysis of AR using STRING, a well-known database of both known and predicted protein–protein interactions (Fig. 5A). Because we have focused on the tumor-suppressive function of AR, NKX3.1, a transcriptional repressor (Combined Score: 0.981) was identified (Fig. 5A). We then analyzed the public ChIP-seq database (ChIP-Atlas) and identified colocalization of AR and NKX3.1 at the promoter region of NKX3.1 (Fig. 5B). These results imply that the AR regulates transcription of the target gene in combination with NKX3.1, a coregulator of AR, as previously reported [33, 34]. It has been reported that YAP1, a transcription factor regulated by the Hippo pathway, interacts with AR and contributes to the growth in PCa [35]. When we analyzed NCBI GEO data set (GSE6919) of normal prostate tissues, prostate tumor tissues, and metastatic prostate tumor tissues in PCa patients, the mRNA expression of YAP1 was significantly increased in metastatic prostate tumor tissues compared to primary ones (Fig. 5C). In contrast, expression of NKX3.1 was reduced in metastatic tissues compared to primary ones (Fig. 5C). Immunoprecipitation analyses were performed to evaluate if YAP1 or NKX3.1 was involved in the anti-migratory action of GV1001. The protein level of YAP1 binding to AR was reduced by GV1001 treatment in LNCaP cells (Fig. 5D). On the contrary, the binding of NKX3.1 with AR was sharply increased by GV1001 (Fig. 5D), and western blot analyses further indicated that the total protein expression of NKX3.1 was increased by GV1001 in LNCaP cells (Fig. 5E). Subcellular fractionation also showed the cytoplasmic retention and the subsequent nuclear loss of YAP1 in GV1001-exposed cells (Fig. 5F). Immunocytochemistry further confirmed the localization of total and Ser-127 phosphorylated YAP1 in the cytoplasm (Fig. 5G). Hippo pathway-induced YAP1 phosphorylation eventually leads to YAP/TAZ ubiquitination and degradation [36]. We also found that GV1001 accelerated the ubiquitination of YAP1 in LNCaP cells (Fig. 5H). To test whether GV1001-stimulated Gαs-cAMP signaling affects the phosphorylation of YAP1, we used adenylate cyclase inhibitors, KT5720 and KH7. GV1001-induced YAP1 phosphorylation was attenuated by treatment with KT5720 (10 μM) or KH7 (5 μM) (Fig. 5I). We then determined mRNA expressions of AR target genes, AR, PSA, and NKX3.1, and a representative YAP target gene, CTGF using quantitative PCR analyses. We revealed that the mRNA expression of AR target genes, AR, PSA, and NKX3.1, was highly upregulated by 10 μM GV1001 (Fig. 5J). In contrast, GV1001 potently reduced the mRNA level of CTGF (Fig. 5J). These data indicate that GV1001-stimulated Gαs signaling evokes both inhibition of YAP1 transcriptional activity and upregulation of AR transcriptional activity via common cAMP/PKA pathway. We further assessed promoter binding activities of the AR complex using ChIP analyses (Fig. 5K). AR complex enrichments at the promoter region of AR target genes, PSA and NKX3.1, were significantly enhanced by GV1001 treatment (Fig. 5K). However, AR complex enrichment at the CTGF gene promoter, a representative YAP1 target gene, was decreased by GV1001 (Fig. 5K). Collectively, these results suggest that GV1001 inactivates YAP1 via Gαs-cAMP signaling and subsequently suppresses the AR interaction with YAP1, and consequently switches AR-coregulator to NKX3.1.

### Involvement of YAP1 in the migration and proliferation of PCa cells

**Fig. 6:**
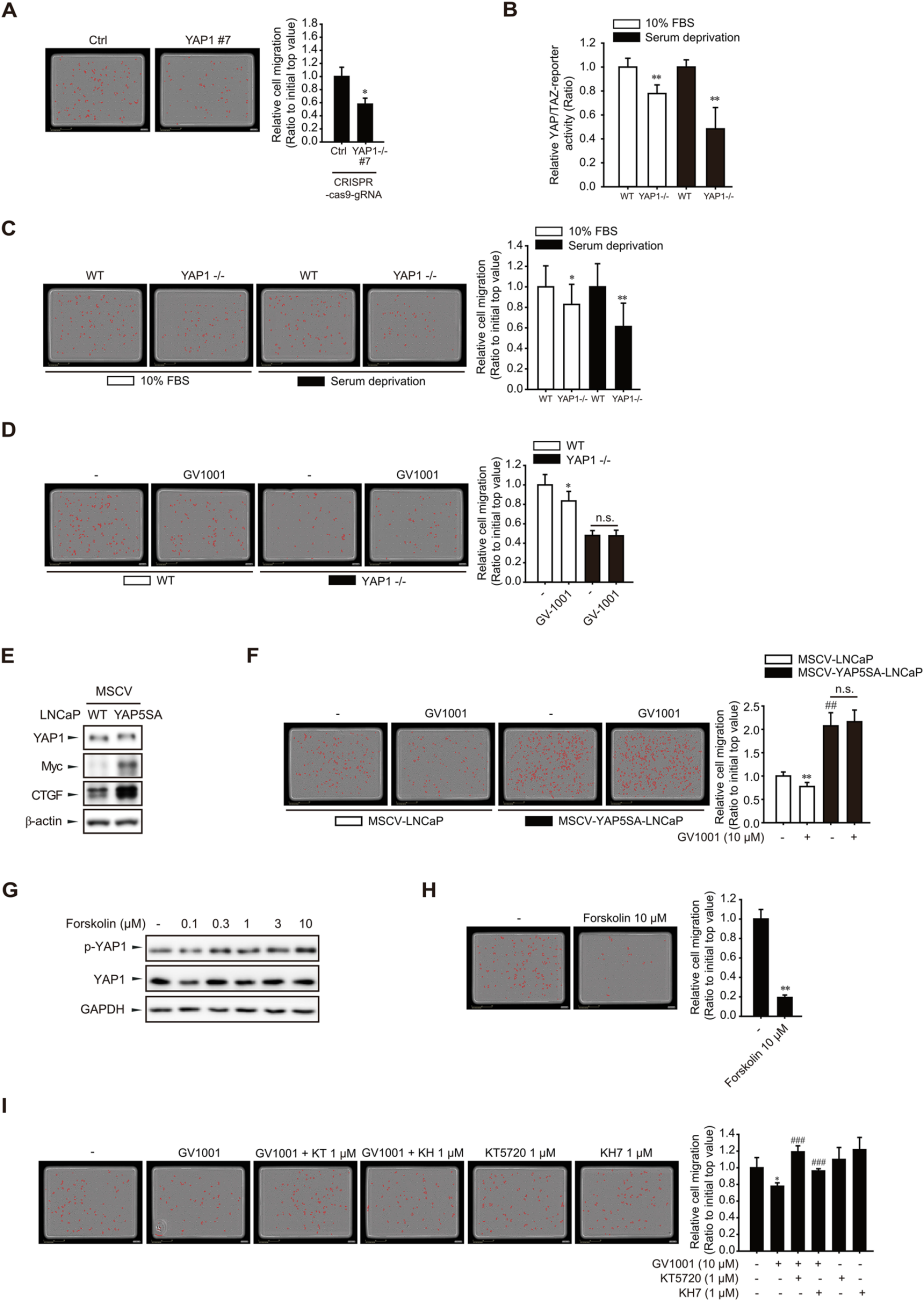
Involvement of YAP1 in LNCaP cell migration. **A** Migration of YAP1 knockout LNCaP cells. Migratory cell number was determined by IncuCyte ZOOM live-cell analysis system. Data represent means ± SD (n = 4, *P < 0.05 vs control cell). **B** YAP/TAZ-reporter activity of YAP knockout LNCaP cells. Control and YAP knockout LNCaP cells were transiently transfected with YAP/TAZ-reporter plasmid (TEAD reporter, 1 μg/ml) and the cells were incubated with or without 10% FBS for 24 h. Luciferase activity was determined to assess YAP/TAZ-mediated gene transcription. Data represent means ± SD (n = 6, **P < 0.01 vs control LNCaP cells). **C** Diminished cell migration by YAP knockout in serum-containing and serum-deprived conditions. Data represent means ± SD (n = 4, *P < 0.05, **P < 0.01 vs vehicle-treated control). **D** Effect of GV1001 on YAP1 knockout LNCaP cell migration. Control- and YAP1-knockout LNCaP cells were treated with 10 μM GV1001 for 24 h, and cell migration was quantified by IncuCyte ZOOM live-cell analysis system. Data represent means ± SD (n = 4, *P < 0.05 vs vehicle-treated control). **E** Establishment of constitutive active YAP5SA-LNCaP cells. Protein expression of YAP1, Myc, and CTGF were determined by immunoblot analyses. **F** Effect of GV1001 on the migration of YAP5SA-overexpressing LNCaP cells. Control and YAP-5SA cells were exposed to 10 μM GV1001 for 24 h. Data represent means ± SD (n = 4, **P < 0.01 vs vehicle-treated control, ^##^P < 0.01 vs control-LNCaP cell group). **G** Forskolin-induced YAP1 phosphorylation. LNCaP cells were treated with 0.1–10 μM Forskolin for 1 h, and protein levels of YAP1 and p-YAP1 were determined by immunoblot analysis. **H** Effect of forskolin on LNCaP cell migration. LNCaP cells were treated with forskolin (10 μM) for 24 h. Data represent means ± SD (n = 4, **P < 0.01 vs vehicle-treated control). **I** Reversal of GV1001-induced anti-migratory activity by KT5720 and KH7. LNCaP cells were incubated with 10 μM GV1001 in the presence or absence of KT5720 (1 μM) or KH7 (1 μM) for 24 h. Data represent means ± SD (n = 4, *P < 0.05, **P < 0.01 vs vehicle-treated control, ^###^P < 0.005 vs GV1001-treated group)

To further define whether GV1001-mediated YAP1 inactivation is related to the reduced PCa cell migration, YAP1 knockout LNCaP cells were established by CRISPR/Cas9 gene editing. Although we did not identify any significant changes in YAP1 protein levels in all clones (Additional file 1: Fig. S1A), T7E1 analysis showed that the YAP1 gene was edited in YAP1 knockout #7 clone (Additional file 1: Fig. S1B). Moreover, the cell migration intensity of the YAP1 knockout #7 clone (LNCaP-YAP1 sgR/Cas9 cells) was diminished compared to negative control-transfected LNCaP cells (LNCaP-control sgR/Cas9 cells) (Fig. 6A). YAP/TAZ-responsive reporter gene assay using TEAD promoter confirmed that the YAP1-dependent gene transcription was reduced in LNCaP-YAP1 sgR/Cas9 cells both in the presence or absence of serum (Fig. 6B). The migratory capacity of LNCaP-YAP1 sgR/Cas9 cells was also decreased both in the presence or absence of serum (Fig. 6C) We then tested the effects of GV1001 on the cell migration of YAP1 knockout cells. Exposure of LNCaP-control sgR/Cas9 cells to 10 μM GV1001 suppressed the cell migration, but the LNCaP-YAP1 sgR/Cas9 cells did not show any significant response in the presence of GV1001 (Fig. 6D). We then established LNCaP cells expressing mutant YAP1 in which the serine residue targeted by LATS1/2 and necessary for cytoplasmic retention are substituted with alanine (YAP5SA) (Fig. 6E)[37]. Notably, the transwell migration assay demonstrated that the basal cellular migration capacity of LNCaP-YAP5SA cells was increased (Fig. 6F). However, GV1001 treatment (10 μM) did not inhibit cell migration of LNCaP-YAP5SA cells expressing constitutively active YAP1 (Fig. 6F). Thus, the anti-migratory effect of GV1001 seems to be related to the suppression of Hippo pathway-dependent YAP1 activation. To check if cAMP/PKA activation by GV1001 is essential for the YAP1 phosphorylation and subsequent inactivation, we further used an adenylate cyclase activator, forskolin. Forskolin (0.3–10 μM) increased Ser-127 phosphorylation of the YAP1 (Fig. 6G). Moreover, forskolin also suppressed the LNCaP cell migration (Fig. 6H). We further confirmed that the Gαs-cAMP signaling inhibitors, KT5720 (1 μM) and KH7 (1 μM), restored the GV1001-induced inhibition of cell migration (Fig. 6I). Taken together, these findings support the concept that GV1001 phosphorylates YAP1 via Gαs-cAMP-mediated hippo pathway activation and inhibits the migration and metastasis of PCa cells.

## Discussion

Ligand-stimulated GnRHR triggers multiple signaling pathways through diverse Gα proteins. Among the G protein pathways, the activated Gαq mediates reproductive function in pituitary cells and is considered as the primary pathway for GnRHR [7–9]. It has been also reported that GnRH analogs possess inhibitory effects in several cancer cell types via Gαi protein. GnRHR ligands-stimulated Gαi activation inhibited cell proliferation of endometrial (HEC-1A and Ishikawa) and ovarian cancer cells (EFO-21 and EFO-27) through the suppression of mitogenic signal transduction [38–42]. However, the Gαs-mediated pharmacological function of the GnRHR ligand is still unclear. In our previous study, we identified GV1001 as a novel GnRHR ligand to selectively activate Gαs/cAMP pathway and demonstrated that the peptide showed an inhibiting effect on tumor growth in LNCaP cells-implanted xenograft model [22]. It has been reported that the anti-proliferation effect of GnRH-II in PCa cells is associated with GnRHR and Gαs/cAMP pathway [43]. However, the precise role of GnRHR-mediated Gαs-cAMP signaling in cancer progression needs to be further clarified. In the present study, we showed for the first time that GV1001 suppressed EMT of PCa cells and cancer cell migration in vitro. In addition, GV1001 potently inhibited liver metastasis of PCa cells implanted in the spleen in vivo.

Because AR plays a pivotal role in the development of PCa, AR targeting by inhibiting either biosynthesis of androgens or ligand-receptor interaction has been the mainstream of current therapeutic intervention for PCa malignancy [44, 45]. However, AR has been suggested as a key negative factor for the EMT process and metastasis of prostate cancer. A series of recent reports have demonstrated that inhibition of AR activity contributes to the cell migration and EMT process of PCa [29, 31, 46].

Here, we also revealed that both AR inactivation and AR deletion promote the migratory capacity of LNCaP cells. Unexpectedly, we found that GV1001 treatment stimulates AR transcriptional activation with an increase in AR phosphorylation in LNCaP cells. GV1001 as a selective ligand for Gαs/cAMP pathway phosphorylates Ser-81 of AR, which is required for the inhibition of EMT and cell migration of LNCaP cells. Conversely, we found that LA, a GnRHR-Gαq activator, inhibits AR phosphorylation via the Gαq-calcium signaling pathway. There is still much that remains unknown about the relationship between AR phosphorylation and Gαs-cAMP signaling. One possible explanation for this is that PKA could phosphorylate not only AR but also HSP90, which is a binding partner of AR. It has been reported that AR phosphorylation in the hinge region, which is critical for nuclear import and transcriptional activity, could be induced by PKA [47]. However, whether PKA directly phosphorylates AR or not is still unclear since this process involves multiple other molecules. Nevertheless, PKA could be potential candidate for mediating AR phosphorylation and transactivation in PCa cells.

Coregulator recruitment is an important step for the control of AR-dependent gene transcription. Depending on the type of coregulator, the transcription of the AR target genes and the cancer progression could be affected [48]. For example, transcription intermediary factor2 (TIF2), a coregulator of AR, is upregulated in response to interleukin-6 and promotes resistance to bicalutamide, an AR antagonist [49]. NKX3.1, a prostate epithelial factor is a homeodomain protein that plays an important role in the advance of PCa [50]. NKX3.1 is known to be one of the target genes of AR activation and inhibit the proliferation and tumorigenesis of PCa [51]. NKX3.1 deficient LNCaP cells showed enhanced cell proliferation, metastasis to the lymphatic system and tumor growth in vivo [52]. Hippo pathway functions as a tumor suppressor signal in normal cells and regulates cell proliferation, apoptosis, and progenitor cell development [53, 54]. The Hippo signaling consists of the mammalian ste2-like kinases (MST1/2) and large tumor suppressor kinase (LATS1/2) in mammals [55]. Activation of the Hippo pathway leads to the degradation of YAP1 after LATS1/2-mediated Ser-127 phosphorylation of YAP1. In an environment where the Hippo pathway is suppressed, the accumulated YAP1 enters the nucleus and promotes the transcription of various oncogenic genes such as *cyr61* and *ctgf* [56]. Overexpression of the YAP1 target genes, including CTGF and Cyr61, is actively involved in cell proliferation, reprogramming, stemness, EMT, anti-apoptosis, and chemoresistance acquisition in cancer cells [54, 57, 58]. Recent studies have identified that YAP1 is a coregulator of AR in PCa cells [35, 59]. Here, we identified that GV1001 treatment inactivated YAP1 through Gαs/cAMP-dependent phosphorylation, vice versa induced protein expression of NKX3.1. Immunoprecipitation and ChIP analyses confirmed that coregulator binding of AR was switched from YAP1 to NKX3.1 in PCa cells exposed to GV1001. The switch of AR coregulators consequentially leads to changes in transcriptional regulation of AR target genes, which seems to be related to inhibition of EMT and cell migration in PCa cells. We have previously shown that GV1001 treatment suppresses proliferation and migration of AR-negative PC-3 cells [22]. Hence, it could be possible that YAP1 inactivation by GV1001 inhibits cell migration in an AR-independent manner.

A recent study reported that the activity of the Hippo-YAP pathway could be controlled by GPCRs coupled with the individual G protein. Selective agonists on GPCRs activating G_12/13_, Gαq, or Gαi protein stimulate the transcriptional activity of YAP1, but Gαs coupled GPCR signaling inhibits YAP/YAZ [60].

## Conclusion

**Fig. 7:**
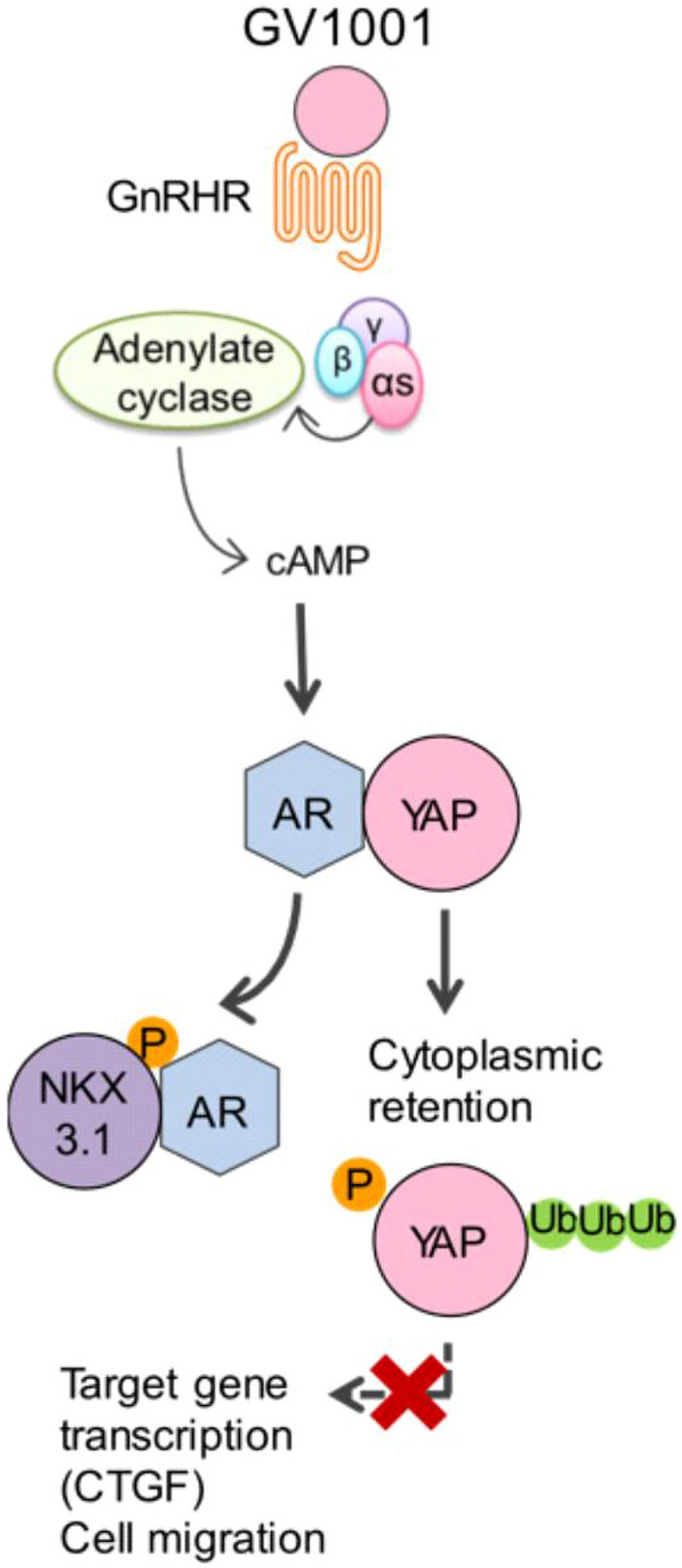
Schematic diagram for anti-metastatic effect of GV1001. GV1001-stimulated GnRHR/Gαs/cAMP pathway simultaneously causes both the activation of AR and the inactivation of YAP via phosphorylation of Ser-81 AR and Ser-127 YAP1, respectively. NKX3.1 upregulation by AR activation and YAP1 degradation in GV1001-treated PCa cells switches AR-YAP1 binding to AR-NKX3.1 binding

Collectively, these observations suggest a new paradigm that GnRHR-driven inactivation of the YAP1-AR axis could be a therapeutic target for prostate cancer. We have previously shown that GV1001 as a GnRHR biased ligand, suppresses testosterone production as well as tumor growth of LNCaP xenograft in vivo [22]. Because GV1001, as a new GnRHR ligand and a YAP1 inhibitor, represents a potential therapeutic for prostate cancer without causing cancer cell migration and metastasis by AR depletion (Fig. 7).

## Supplementary Information

**Additional file 1: Figure S1.** Establishment of YAP1 knockout LNCaP cells. (A) Validation of YAP1 knockout LNCaP cells by CRISPR/Cas9 system. YAP1 expression was determined by immunoblot analysis. All of the results were confirmed by multiple experiments. (B) Validation for CRISPR/Cas9 editing. Representative gel images of T7E1-treated PCR products amplified from the target sites of YAP1. Cleaved-products are designated by arrowheads.

## Data Availability

All data generated or analysed during this study are included in this published article and its supplementary information files.

## Abbreviations

ADT: Androgen deprivation therapy
AR: Androgen receptor
ARE: Androgen-response element
CA: Cetrorelix acetate
CRE: CAMP response element
CRPC: Castration-resistant prostate cancer
DAPI: 4′6-Diamidino-2-phenylindole
DMF: Dimethylformamide
EDC: 1-Ethyl-3-(3-dimethylaminopropyl)-carbodiimide hydrochloride
EDTA: Ethylenediamine tetraacetic acid
EGTA: Ethyleneglycol tetraacetic acid
FITC: Fluorescein isothiocyanate
FSH: Follicle-stimulating hormone
GnRH: Gonadotropin-releasing hormone
GnRHR: Gonadotropin-releasing hormone receptor
HOBt: Hydroxybenzotriazole
HRP: Horseradish peroxidase
hTERT: Human telomerase reverse transcriptase catalytic subunit
LA: Leuprolide acetate
LH: Luteinizing hormone
MMP2: Matrix metalloproteinase-2
MMP9: Matrix metalloproteinase-9
NSCLC: Non-small cell lung cancer
PBS: Phosphate buffered saline
PCa: Prostate cancer
PSA: Prostate-specific antigen
SDS: Sodium dodecyl sulfate
