# Supplementary figures and images for: Anti-metastatic effect of GV1001 on prostate cancer cells; roles of GnRHR-mediated Gαs-cAMP pathway and AR-YAP1 axis
Source: Cell Biosci. 2021 Nov 7;11:191. doi: 10.1186/s13578-021-00704-3 (PMC8574053; doi:10.1186/s13578-021-00704-3)

Additional file 1: Figure S1.

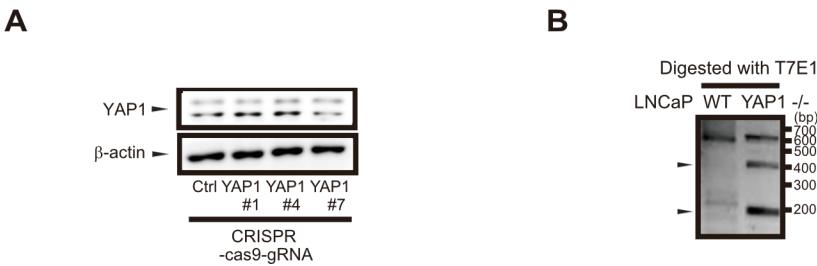

Supplement: Supplementary file 1 — Additional file 1: Figure S1. Establishment of YAP1 knockout LNCaP cells. (A) Validation of YAP1 knockout LNCaP cells by CRISPR/Cas9 system. YAP1 expression was determined by immunoblot analysis. All of the results were confirmed by multiple experiments. (B) Validation for CRISPR/Cas9 editing. Representative gel images of T7E1-treated PCR products amplified from the target sites of YAP1. Cleaved-products are designated by arrowheads. [file 13578_2021_704_MOESM1_ESM.pdf]
